# Elevated levels of serum IL-5 are associated with an increased likelihood of major depressive disorder

**DOI:** 10.1186/1471-244X-12-2

**Published:** 2012-01-09

**Authors:** Antti-Pekka Elomaa, Leo Niskanen, Karl-Heinz Herzig, Heimo Viinamäki, Jukka Hintikka, Heli Koivumaa-Honkanen, Kirsi Honkalampi, Minna Valkonen-Korhonen, Ilkka T Harvima, Soili M Lehto

**Affiliations:** 1 Department of Psychiatry, Kuopio University Hospital and University of Eastern Finland, Kuopio, Finland; 2 Central Hospital of Central Finland, Jyväskylä, and University of Eastern Finland, Kuopio, Finland; 3 Institute of Biomedicine, Division of Physiology and Biocenter Oulu, University of Oulu, Finland; 4 Department of Clinical Medicine, University of Oulu, Finland; 5 Department of Psychiatry, Lapland Hospital District, Rovaniemi, Finland; 6 Kuopio Psychiatric Center, Kuopio, Finland; 7 Department of Dermatology, Kuopio University Hospital and University of Eastern Finland, Kuopio, Finland

## Abstract

**Background:**

Inflammatory mediators in both the peripheral circulation and central nervous system (CNS) are dysregulated in major depressive disorder (MDD). Nevertheless, relatively little is known about the role of the T-helper (Th)-2 effector cytokines interleukin (IL)-5 and IL-13 in MDD.

**Methods:**

We examined the serum levels of these cytokines and a Th-1 comparison cytokine, interferon (IFN)-γ, in 116 individuals (MDD, n = 58; controls, n = 58).

**Results:**

In our basic multivariate model controlling for the effects of potential confounders on the associations between MDD and the examined cytokines, each 1-unit increase in the serum IL-5 level increased the likelihood of belonging to the MDD group by 76% (OR 1.76, 95% CI 1.03-2.99, p = 0.04; model covariates: age, gender, marital status, daily smoking and alcohol use). The likelihood further increased in models additionally controlling for the effects of the use of antidepressants and NSAIDS, and a diagnosis of asthma. No such associations were detected with regard to IL-13 (OR 1.08, 95% CI 0.96-1.22, p = 0.22) or IFN-γ (OR 1.02, 95% CI 0.99-1.05, p = 0.23).

**Conclusions:**

Elevated levels of IL-5, which uses the neural plasticity-related RAS GTPase-extracellular signal-regulated kinase (Ras-ERK) pathway to mediate its actions in the central nervous system (CNS), could be one of the factors underlying the depression-related changes in CNS plasticity.

## 1. Background

According to the monocyte-T-lymphocyte hypothesis of major depression, the pathophysiology of major depressive disorder (MDD) anticipates immune system activation [1]. Cytokines are immune system signaling molecules that affect the synthesis, release, and cell reuptake of monoamines [2]. Both their absence and over-expression affects central nervous system functions such as synaptic plasticity, neurogenesis, and neuromodulation in varying ways, including activity within basal ganglia and the frontal cortex [3-6]. They also modulate the activity of the hypothalamus-pituitary axis (HPA), which is a central factor in the pathogenesis of MDD [7].

Several studies have indicated MDD as an inflammatory state with elevated levels of proinflammatory cytokines [e.g. Interleukin (IL)-6 and -12 and interferon (IFN)-γ] [8]. Cytokines such as IL-1 and tumor necrosis factor (TNF)-α are also dysregulated, and they have been suggested to serve as neuroplasticity-modulating factors in MDD neuropathology [9]. Moreover, much emphasis has been placed on the imbalance between T-helper (Th) cell cytokines Th1 and Th2 in the pathogenesis of MDD [10]. While MDD is linked to disturbance in the homeostasis of immune systems [7,11], it also increases the likelihood of several somatic conditions such as cardiovascular diseases, diabetes, and cancer [12], as well as asthma and other allergic conditions [13]. In particular, asthmatic adolescents have a significantly increased risk of the early development of psychiatric disorders, while MDD patients with comorbid asthma suffer from greater impairments in asthma symptoms [14].

Th cells regulate the immune systems through the production of cytokines [15]. Th cells are mainly divided into two types according to their polarization. Th1 cells regulate cell-mediated immune responses and are characterized by IL-2 and IFN-γ production [16]. IFN-γ has various immunoregulatory actions. Its biological responses are closely integrated with the other cytokine mechanisms, with signaling pathways especially cross-talking with IFN-α [17]. IFN-γ is one of most studied cytokines in MDD [18]. Th2 cells regulate humoral immunity via B-cell actions by producing IL-4, IL-5, and IL-13 [19]. Furthermore, other T-cells such as Th17 and regulatory T-cells have emerged as important factors controlling immune responses and inflammation [20].

Cytokines IL-5 and IL-13 play important roles in the pathophysiology of various autoimmune diseases such as asthma [21] and Graves' disease [22]. IL-4 mainly functions by activating Th2 cells, while IL-5 and IL-13 work as effector cytokines of activated Th2 cells, i.e. they are secreted by Th cells after the cells are activated [23]. Elevated levels of IL-13 and lower levels of IFN-γ have been associated with MDD [24]. Interestingly, possible routes between IL-5 and MDD have also been stated. There is a genetic risk for MDD through the colony stimulating factor 2 receptor β (CSF2RB) haplotype, which encodes a protein in high affinity receptors binding IL-5 [25]. Furthermore, a gene set analysis of post-mortem brain tissue has suggested the upregulation of IL-5 levels in MDD [26]. However, to the best of our knowledge, there have been very few studies examining IL-5 and IL-13 in relation to MDD.

The goal of this study was to examine the roles of the Th2 effector cytokines IL-5 and IL-13 and an established comparison Th1 cytokine, IFN-γ, in MDD. While IL-4 has been intensively studied with regard to MDD, increasing evidence has accumulated of the roles of other Th2 cytokines in contributing to neuromodulatory alterations similar to those observed in MDD (e.g., impaired neuroplasticity). Thus, individuals with MDD would be likely to show elevations in the secretion of these cytokines. IFN-γ was chosen because of its central relationship with MDD, and due to its comparative role to the Th2 cytokines as a Th1 cytokine.

## 2. Methods

The study was conducted as part of the Kuopio Depression (KUDEP) four-phase general population study involving adults aged 25-64 years in the province of Kuopio in Eastern Finland. A random sample of 3004 participants was selected in 1998 from the National Population Register and was followed up in 1999 and 2001. The baseline sample included 2050 participants, the first follow-up sample (1999) 1722 participants, and the second follow-up sample (2001) 1593 participants. The second follow-up questionnaire was only sent to those subjects who responded in either of the preceding surveys. Questionnaires were re-sent to the non-respondents one month later after each follow-up. Participants responded to questions concerning their age, gender, marital status (married or living with a partner vs. living alone), asthma diagnosis by a physician (yes vs. no), daily smoking (yes vs. no), alcohol use (≥ 2 times per week vs. less), and the use of antidepressant medications (yes vs. no).

In 2005, a sub-sample of 427 participants from the previous study phases reporting either high or low levels of adverse mental symptoms were asked to take part in a clinical evaluation and laboratory testing [27,28] as a fourth study phase. For the present study, we selected those participants whose Beck Depression Inventory (BDI) [29] scores indicated depressive symptoms (BDI scores ≥ 10; depressed) or a euthymic state (BDI scores ≤ 9; controls) consistently at all three earlier data collection points. The mean BDI scores (standard deviation, SD) for the depressed (n = 58) were 18.1 (9.1) in 1998, 21.5 (9.9) in 1999, and 20.9 (11.1) in 2001. The corresponding means (SD) for the controls (*n *= 58) were 2.6 (2.2) in 1998, 2.9 (2.6) in 1999, and 2.1 (1.9) in 2001.

Psychiatric interviews were conducted for all participants, including the controls, by a trained, experienced research nurse using the Structured Clinical Interview for DSM-IV (SCID-I and SCID-II) [30-32]. Additionally, the 29-item Hamilton Depression Rating Scale (HAM-D-29) [33] was used for the assessment of depression severity. The HAM-D-29 includes the 21-item Hamilton Depression Rating Scale (HAM-D-21) with a supplementary 8-item subscale (Atypical Depression Supplement, ADS) for measuring atypical features of depression. The ADS includes questions regarding weight gain, increased appetite/eating, carbohydrate craving, hypersomnia, leaden paralysis, and social withdrawal. Subjects with bipolar disorders were excluded and the primary diagnosis for all the rest of the depressed participants was MDD. Data concerning the participants' somatic medications were acquired from the nationwide Social Insurance Institute. Approval for the study was obtained from the Ethics Committee of Kuopio University Hospital and the University of Eastern Finland. The study protocol was in accordance with the Declaration of Helsinki. All participants provided written informed consent before entering the study.

After the clinical interviews, the participants were given a referral to the laboratory, and were then instructed to visit the laboratory at the latest during the following week. The body mass index (kg/m^2^) was calculated from height and body weight measured in light clothing without shoes. The venous blood serum samples for the cytokine analyses were stored at -80°C until run. IL-5 (pg/mL), IL-13 (pg/mL) and IFN-γ (pg/mL) levels were analyzed by multiplexing with Bio-Plex Human Cytokine Panel 1 utilizing a Bio-Plex 200 instrument based on Luminex xMAP technology (Bio-Rad Laboratories Inc., CA, US). The Luminex method and the standard enzyme-linked immunosorbent assay (ELISA) are highly correlated [34-36]. Before analyses, the samples were centrifuged for 15 min at 3000 rpm, and diluted 1:2 in a sample matrix. The samples were assayed singly, and the assay conditions were standardized and pre-optimized to ensure optimal reproducibility of the assays. The kit instructions and instrument manuals were followed accordingly. The intra-assay and interassay variation for the kit analyses were 4.6-13.8% and 3.7-17.2%, respectively. The results were calculated with BioPlex Manager Software version 4.3 with five-parameter logistic equations [37]. High sensitivity range standard settings were utilized. The values between zero (blank sample) and the lowest standard (IL-5: 0.52 pg/ml; IL-13: 0.71 pg/ml; IFN-γ: 0.30 pg/ml) were extrapolated from the standard curve by the software. In statistical analyses, the non-detectable samples (ND) were marked as the mean value between zero and the lowest extrapolated value of each cytokine [38]. There were 17 NDs for IL-5, 30 for IL-13, and 52 for IFN-γ.

The data were analyzed using SPSS version 17.0 statistical software (SPSS Inc., Chicago, IL). Differences between the MDD and control groups were assessed using the chi-squared test for categorical variables, and the Student's t-test and the Mann-Whitney U-test for continuous variables. Parametric tests were used with normally distributed and non-parametric tests with non-normally distributed variables. The differences between the MDD and control groups in the examined cytokines were further examined with logistic regression modeling. The modeling was performed in two steps. First, we formed a basic model consisting of general parameters either showing differences between the groups or known to affect the levels of IL-5, IL-13 or IFN-γ (Model 1: age, gender, marital status, alcohol use [39], and daily smoking [40]). Secondly, for more comprehensive evaluation of the effects of potential confounders, we formed models that included the variables in Model 1 together with further adjustments for one of the following covariates: the use of antidepressants (Model 2), the use of NSAIDs (Model 3), and a diagnosis of asthma (Model 4). The cytokines were inserted into the models as continuous variables. The odds ratios (OR) show the likelihood for each 1-point increase in the serum levels of these cytokines, i.e. OR show the odds for a covariate belonging to the MDD group in relation to the control group.

## 3. Results

**Figure 1:**
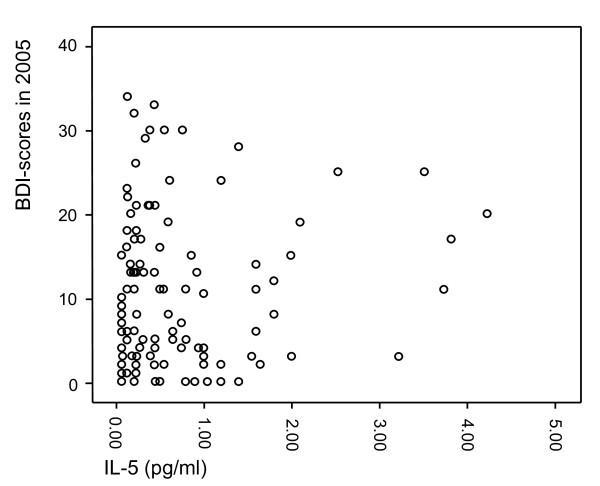
**Scatterplot of IL-5 levels in relation to BDI scores from the 2005 questionnaire**.

**Figure 2:**
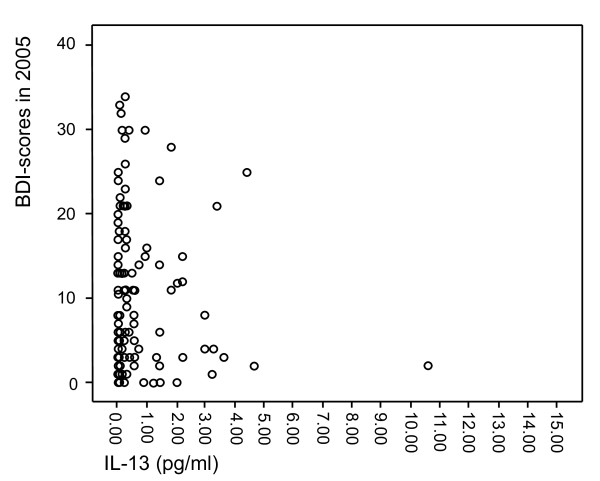
**Scatterplot of IL-13 levels in relation to BDI scores from the 2005 questionnaire**.

**Figure 3:**
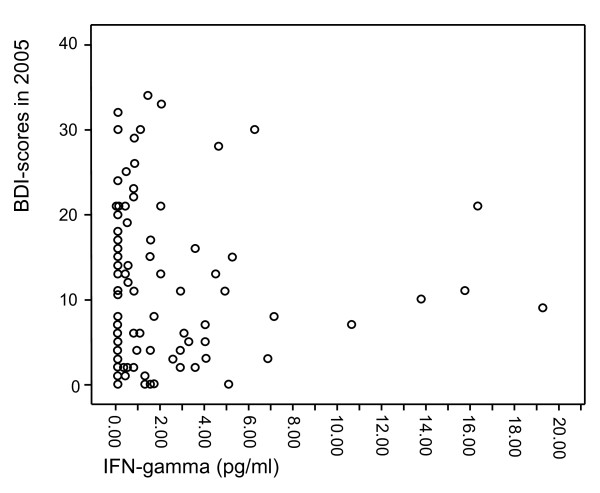
**Scatterplot of IFN-γ levels in relation to BDI scores from the 2005 questionnaire**.

The characteristics of the study population are presented in Table 1. The individuals in the MDD group more often lived alone, smoked, and used antidepressant medication. They also showed higher scores on all the depression scales used in the present study, i.e. HAM-D-21, HAM-D-29, ADS, and BDI. The levels of IL-5, IL-13 and IFN-γ did not significantly differ between the groups in the crude analysis. Moreover, scatterplots were formed to visualize the relationships between IL-5 (Figure 1), IL-13 (Figure 2) and IFN-γ (Figure 3) and the BDI scores from the 2005 questionnaire.

**Table 1:** The characteristics of the study participants.

	MDD	Controls		
	n = 58	n = 58	Statistics*	p-value
Age	53.40 ± 1.22	53.21 ± 1.20	t = -0.011	0.912^a^
Female, n (%)	40 (69.0)	40 (69.0)	χ^2 ^= 0.00	1.000^b^
Married or living with a partner, n (%)	48 (82.5)	55 (94.8)	χ^2 ^= 5.25	0.039^b^
Daily smoking, n (%)	18 (31.0)	4 (6.9)	χ^2 ^= 10.99	0.001^b^
Alcohol use, ≥ 2 times per week (%)	9 (15.5)	10 (17.2)	χ^2 ^= 0.06	0.802^b^
Asthma, n (%)	3 (5.2)	5 (8.6)	χ^2 ^= 0.537	0.464^b^
BMI (kg/m^2^)	27.24 ± 0.86	27.13 ± 0.65	Z = -0.04	0.967^c^
HAM-D-21	12.00 ± 0.73	2.98 ± 0.36	Z = -8.00	< 0.001^c^
ADS	2.84 ± 0.43	0.59 ± 0.13	Z = -4.04	< 0.001^c^
HAM-D-29	14.84 ± 0.99	3.57 ± 0.41	Z = -7.86	< 0.001^c^
BDI	17.68 ± 0.97	3.55 ± 0.41	Z = -8.77	< 0.001^c^
Use of NSAIDs, n (%)	7 (12.1)	1 (1.7)	--	0.061^d^
Use of oral corticosteroids, n (%)	5 (8.6)	3 (5.2)	--	0.717^d^
Use of antidepressants, n (%)	22 (37.9)	4 (6.9)	χ^2 ^= 16.06	< 0.001^b^
IL-5 (pg/ml)	0.81 ± 0.13	0.54 ± 0.08	Z = -1.87	0.061^c^
IL-13 (pg/ml)	7.51 ± 6.05	0.89 ± 0.22	Z = -0.60	0.549^c^
IFN-γ (pg/ml)	12.03 ± 5.96	3.03 ± 0.91	Z = -0.43	0.664^c^
IFN-γ/IL-5 ratio	6.97 ± 1.99	24.69 ± 11.16	Z = -0.39	0.697^c^

Values are means ± standard errors unless otherwise stated.

^a ^Student's t-test; ^b ^Chi-squared test; ^c ^Mann-Whitney U-test; ^d ^Fisher's exact test; * Where applicable

Abbreviations: ADS = atypical depression supplement; BMI = body mass index, BDI = Beck Depression Inventory, HAM-D = Hamilton Depression Rating Scale, IL = interleukin, MDD = major depressive disorder; NSAID = non-steroidal anti-inflammatory drug

In the multivariate models (Table 2) controlling for the effects of potential confounders on the associations between depression and the examined cytokines, each 1-unit increase in the serum IL-5 levels increased the likelihood of belonging to the MDD group by 76% (OR 1.76, 95% CI 1.03-2.99, p = 0.038) in Model 1 adjusted for age, gender, marital status, daily smoking, and alcohol use. The observations remained essentially similar in the other models further adjusted for the use of antidepressants (Model 2: OR 2.06, 95% CI 1.16-3.63, p = 0.013), the use of NSAIDS (Model 3: OR 1.89, 95% CI 1.10-3.27, p = 0.022) and a diagnosis of asthma (Model 4: OR 1.83, 95% CI 1.07-3.14, p = 0.028). No such associations were observed for IL-13 or IFN-γ (Table 2).

**Table 2:** The likelihood for belonging to the major depressive disorder group.

		Age			Gender			Marital status			Daily smoking			Alcohol use			Cytokine*			Additional covariate (antidepressant/NSAIDs/asthma)		
	
	Model	OR	P	CI95_%_	OR	p	CI95_%_	OR	p	CI95_%_	OR	p	CI95_%_	OR	p	CI95_%_	OR	p	CI95_%_	OR	p	CI95_%_
IL-5*	1.^a^	1.03	0.24	0.98-1.08	1.37	0.49	0.56-3.35	4.05	0.67	0.91-17.96	7.95	0.001	2.34-27.03	1.62	0.40	0.52-5.04	1.76	0.04	1.03-2.99	-	-	-
	2.^b^	1.01	0.69	0.96-1.06	1.51	0.41	0.56-4.06	4.23	0.85	0.82-21.76	10.59	0.001	2.87-39.15	2.32	0.18	0.68-7.88	2.06	0.013	1.16-3.64	11.98	0.001	3.25-44.16
	3.^c^	1.04	0.15	0.99-1.09	1.30	0.58	0.52-3.30	3.99	0.076	0.87-18.39	9.08	0.001	2.60-31.73	1.94	0.29	0.57-6.53	1.89	0.022	1.10-3.27	0.084	0.033	0.01-0.82
	4.^d^	1.03	0.24	0.98-1.08	1.31	0.56	0.53-3.25	3.80	0.81	0.85-17.01	8.60	0.001	2.46-30.09	1.67	0.38	0.54-5.17	1.83	0.028	1.07-3.14	2.45	0.30	0.45-13.43

IL-13*	1^.a^	1.03	0.23	0.98-1.08	1.26	0.61	0.52-3.03	4.50	0.044	1.04-19.50	7.51	0.001	2.22-25.33	1.47	0.50	0.48-4.50	1.08	0.22	0.96-1.22	-	-	-
	2.^b^	1.01	0.69	0.96-1.06	1.30	0.59	0.50-3.36	4.49	0.066	0.91-22.24	9.24	0.001	2.54-33.58	2.02	0.26	0.60-6.84	1.06	0.33	0.94-1.20	0.098	0.001	0.027-0.35
	3.^c^	1.04	0.15	0.99-1.09	1.19	0.71	0.48-2.94	4.35	0.054	0.97-19.47	8.36	0.001	2.41-29.07	1.70	0.39	0.51-5.63	1.09	0.186	0.96-1.24	0.095	0.039	0.10-0.89
	4.^d^	1.03	0.22	0.98-1.08	1.20	0.69	0.49-2.91	4.24	0.055	0.97-18.54	8.18	0.001	2.35-28.52	1.47	0.50	0.48-4.50	1.10	0.14	0.97-1.24	2.65	0.29	0.44-15.87

IFN-γ*	1.^a^	1.03	0.21	0.98-1.08	1.32	0.54	0.55-3.19	3.92	0.069	0.90-17.09	7.41	0.001	2.19-25.14	1.61	0.42	0.51-5.05	1.02	0.23	0.99-1.05	-	-	-
	2.^b^	1.01	0.66	0.96-1.06	1.31	0.57	0.51-3.40	3.86	0.094	0.80-18.74	8.95	0.001	2.46-32.47	2.15	0.23	0.62-7.41	1.01	0.22	0.99-1.04	0.10	0.001	0.029-0.37
	3.^c^	1.04	0.15	0.99-1.09	1.22	0.67	0.49-3.02	4.18	0.062	0.93-18.85	8.04	0.001	2.32-27.83	1.74	0.37	0.52-5.80	1.02	0.25	0.99-1.05	0.11	0.053	0.011-1.03
	4.^d^	1.03	0.21	0.98-1.08	1.28	0.59	0.53-3.11	3.77	0.078	0.86-16.49	7.63	0.001	2.23-26.16	1.61	0.41	0.52-5.05	1.02	0.23	0.99-1.05	1.72	0.52	0.33-8.88

^a ^Covariates for Model 1: gender, age, marital status, daily smoking, and alcohol use

^b ^Covariates for Model 2: gender, age, marital status, daily smoking, alcohol use, and antidepressant use

^c ^Covariates for Model 3: gender, age, marital status, daily smoking, alcohol use, and NSAIDs use

^d ^Covariates for Model 4: gender, age, marital status, daily smoking, alcohol use, and diagnosis of asthma

## 4. Discussion

### 4.1 Main findings

The elevation of serum levels of IL-5 was associated with an increased likelihood for belonging to the group with MDD. No such associations were detected with regard to IL-13 or IFN-γ.

### 4.2 Previous literature

Previous data on the connections between IL-5 and MDD are scarce. However, a recent post-mortem gene set analysis suggested an up-regulation of IL-5 levels in the frontal cortices of individuals with MDD [26]. Our findings were consistent with this observation, demonstrating similar elevations in circulating levels of IL-5. This is also in line with studies showing elevated levels of other Th2 cytokines, such as IL-4 and IL-13 [41]. On the other hand, a study by Simon et al. (2008) [42] revealed no difference in IL-5 levels between the MDD group and controls. These findings, however, were not adjusted for potential confounders such as smoking, which appears to lower the levels of serum IL-5 [40]. Thus, the lack of adjustment for smoking in the study by Simon et al., as well as in our study with respect to our initial cross-sectional association (Table 1), may underlie our observation of only borderline significance for elevated IL-5 levels in MDD.

Elevated levels of IL-13, IFN-γ, and the Th1/Th2 ratio have been detected in MDD [19]. In our study, the individuals with MDD had higher overall levels of both IL-13 and IFN-γ, but this difference did not reach statistical significance. However, the small study population of the present study may have increased the risk of type II statistical errors, leading to the lack of detection of an actual difference in the cytokine levels between the study groups. Moreover, the studies of Pavón et al. (2006) [24] and Hernández et al. (2008) [41] represented patient populations and not a random cohort from a general population, and were thus more severely depressed. The mean HAM-D-21 score for the depressed group reported by Hernández et al. was 20.3, and the HAM-D-17 score in the study of Pavón et al. was 24.2, whereas in our MDD group the mean HAM-D-21 score was 12.0. Thus, cytokine differences are likely to be less pronounced in a population-based sample consisting of individuals with a depression score lower than those observed in patient samples.

In this study there was no significant relationship between MDD and asthma. Although there is very little data covering the potential underlying mechanisms connecting asthma and MDD, Th2 cytokines appear to play a role in the pathophysiology of both of these diseases [25,26,43], as asthma and other similar inflammatory diseases have been shown to increase the risk and symptoms of MDD [14].

### 4.3 Possible mechanisms

Peripheral cytokines can cross the blood-brain barrier, influencing complex brain functions both directly and indirectly [12,44]. Pavón et al. (2006) [24] hypothesized that the elevated circulatory cortisol levels associated with MDD could lead to abnormal patterns of synthesis and secretion of Th2 cytokines, thus provoking the depressive symptoms. This may involve several possible mechanisms.

Relatively little is known about IL-5. Most previous studies have not detected alterations in the levels IL-5 of MDD patients [42]. However, IL-5 activates several interesting signaling pathways, with activation of Januse kinase 2 (JAK) 2 and STAT5 being the most essential. The JAK-STAT pathways play important roles in cell proliferation and survival in both the CNS and the periphery through specific and diverse effects on cell responses to hormones, growth factors and cytokines [45,46], while its activation can lead to depressive effects through glucocorticoid signaling [47]. IFN-α and -γ have also been shown to activate JAK family kinases and consequently phosphorylate STATs [48], and further induce depressive symptoms [49]. In addition to JAK-STAT pathways, IL-5 uses the Ras GTPase-extracellular signal-regulated kinase (Ras-ERK) pathway. IL-5 can activate the Ras-ERK pathway [50], which controls neural stem cell functions and maintains specific brain structures [51]. Hyperactivity of Ras-ERK pathways has been observed to cause deficits in synaptic plasticity and hippocampus-related learning in mice [52]. Such mechanisms may play a role in depression. Thus, the elevated levels of IL-5 could be one of the factors inducing a depression-related decrease in central nervous system plasticity.

### 4.4 Strengths and limitations

One of our strengths in this study was the comprehensive evaluation of depressive symptomatology. In evaluating and diagnosing MDD, we used the Structured Clinical Interview for DSM-IV, as well as self-reported and interviewer-rated depression scales. Moreover, several factors such as antidepressant use [53], lifestyle factors [39,40], and various somatic medications and diseases such as asthma [14] affect the levels of cytokines expressed in circulation. Nevertheless, we were able to examine the effects of these potential confounders on our findings, even though the somatic conditions were self-reported. The depressed subjects in the present study suffered homogeneously from MDD, while those with bipolar disorder were excluded. The study population also solely consisted of individuals of Finnish descent, thus minimizing the potential confounding effect of genetic variation on our findings. The main limitation of our study was the small sample size, increasing the risk of type II statistical errors, which might have resulted in the lack of detection of actual significant differences between the groups. The high interassay variation in analysis could have also interfered with the ability to detect differences in cytokine levels. Secondly, due to the cross-sectional study design, we were unable to assess causality or confirm previous suggestions of possible biological routes. A fairly large number of conditions affect serum IL-5 levels, and despite statistical adjustment for known confounders, there remains the potential for residual confounding. Thirdly, we did not have data on some somatic conditions related to the secretion of some of the measured cytokines (e.g. allergic rhinitis, atopic dermatitis), which may have affected our findings. IL-13 may be associated with allergic rhinitis when the individuals are provoked with an allergen [54]. However, some data are available suggesting that the levels of IL-5 and IFN-γ are unaffected even in the presence of an allergen [54]. Furthermore, to the best of our knowledge, these conditions are not clearly associated with MDD; thus, it is unlikely that these individuals would specifically concentrate in the MDD group. Lastly, a general guideline indicates that the number of non-detectable samples can be considered small when they comprise less than 30% of the total sample size [38]. In this study, the proportions of non-detectable samples for IL-5 and IL-13 were below the 30% limit, but IFN-γ exceeded this limit. However, since all the imputed values were close to zero, the effect of the imputation method on the total variance is likely to have been very small.

## 5. Conclusions

We have been able to demonstrate that an increased likelihood of MDD was associated with elevated levels of IL-5. Elevated IL-5, which uses the neural plasticity-related RAS-ERK pathway to mediate its actions in the CNS, could be one of the factors underlying depression-related changes in CNS plasticity. Further studies are needed to more thoroughly understand the exact biological mechanisms through which IL-5 is linked to depression

## Competing interests

The authors declare that they have no competing interests.

## Authors' contributions

A-PE analyzed and interpreted the data, and drafted the manuscript. LN participated in the conception and design of the study and interpreted the data. K-HH, HV, JH, KH, and HK-H participated in the conception of the study, and the acquisition and interpretation of the data. MV-K and ITH participated in the conception of the study and data interpretation. SML participated in the conception and design of the study, analyzed the data and supervised the drafting of the manuscript. All the authors have revised the manuscript for important intellectual content, and have read and approved the final manuscript.

## Pre-publication history

The pre-publication history for this paper can be accessed here:

http://www.biomedcentral.com/1471-244X/12/2/prepub

## References

[B1] Maes M Smith R Scharpe S The monocyte-T-lymphocyte hypothesis of major depression Psychoneuroendocrinology 1995 20 111 116 10.1016/0306-4530(94)00066-J 7899532

[B2] Nadjar A Bluthé RM May MJ Dantzer R Parnet P Inactivation of the cerebral NFkappaB pathway inhibits interleukin-1beta-induced sickness behavior and c-Fos expression in various brain nuclei Neuropsychopharmacology 2005 30 1492 1499 10.1038/sj.npp.1300755 15900319

[B3] Capuron L Pagnoni G Demetrashvili MF Lawson DH Fornwalt FB Woolwine B Berns GS Nemeroff CB Miller AH Basal ganglia hypermetabolism and symptoms of fatigue during interferon-alpha therapy Neuropsychopharmacology 2007 32 2384 2392 10.1038/sj.npp.1301362 17327884

[B4] Capuron L Pagnoni G Demetrashvili M Woolwine BJ Nemeroff CB Berns GS Miller AH Anterior cingulate activation and error processing during interferon-alpha treatment Biol Psychiatry 2005 58 190 196 10.1016/j.biopsych.2005.03.033 16084839 PMC1366492

[B5] McAfoose J Baune BT Evidence for a cytokine model of cognitive function Neurosci Biobehav Rev 2009 33 355 366 10.1016/j.neubiorev.2008.10.005 18996146

[B6] Nelson T Ur C Gruol D Chronic interleukin-6 exposure alters electrophysiological properties and calcium signaling in developing cerebellar purkinje neurons in culture J Neurophysiol 2002 88 475 486 12091569 10.1152/jn.2002.88.1.475

[B7] Pace TWW Hu F Miller AH Cytokine-effects on glucocorticoid receptor function: Relevance to glucocorticoid resistance and the pathophysiology and treatment of major depression Brain Behav Immun 2007 21 9 19 10.1016/j.bbi.2006.08.009 17070667 PMC1820632

[B8] Irwin MR Miller AH Depressive disorders and immunity: 20 years of progress and discovery Brain Behav Immun 2007 21 374 383 10.1016/j.bbi.2007.01.010 17360153

[B9] Khairova RA Machado-Vieira R Du J Manji HK A potential role for pro-inflammatory cytokines in regulating synaptic plasticity in major depressive disorder Int J Neuropsychopharmacol 2009 12 561 578 10.1017/S1461145709009924 19224657 PMC2771334

[B10] Myint AM Leonard BE Steinbusch HW Kim YK Th1, Th2, and Th3 cytokine alterations in major depression J Affect Disord 2005 88 167 173 10.1016/j.jad.2005.07.008 16126278

[B11] Zorrilla EP Luborsky L McKay JR Rosenthal R Houldin A Tax A McCorkle R Seligman DA Schmidt K The relationship of depression and stressors to immunological assays: A meta-analytic review Brain Behav Immun 2001 15 199 226 10.1006/brbi.2000.0597 11566046

[B12] Raison CL Capuron L Miller Ah Cytokines sing the blues: Inflammation of the pathogenesis of major depression Trend Immunol 2006 27 24 31 10.1016/j.it.2005.11.006 PMC3392963 16316783

[B13] Timonen M Jokelainen J Hakko H Silvennoinen-Kassinen S Meyer-Rochow VB Herva A Rasanen P Atopy and depression: results from the Northern Finland 1966 birth cohort study Mol Psychiatry 2003 8 738 744 10.1038/sj.mp.4001274 12888802

[B14] Katon W Asthma, suicide risk and psychiatric comorbidity Am J Psychiatry 2010 167 1020 1022 10.1176/appi.ajp.2010.10050657 20826851

[B15] Parkin J Cohen B An overview of the immune system Lancet 2001 357 1777 1789 10.1016/S0140-6736(00)04904-7 11403834

[B16] Hendrix S Nitsch R The role of T helper cells in neuroprotection and regeneration J Neuroimmunol 2007 184 100 112 10.1016/j.jneuroim.2006.11.019 17198734

[B17] Schroder K Hertzog PJ Ravasi T Hume DA Interferon-gamma: an overview of signals, mechanisms and functions J Leukoc Biol 2004 75 163 189 14525967 10.1189/jlb.0603252

[B18] Kim YK Na KS Shin KH Jung HY Choi SH Kim JB Cytokine imbalance in the pathophysiology of major depressive disorder Prog Neuro-Psychopharmacol Biol Psychiatry 2007 31 1044 1053 10.1016/j.pnpbp.2007.03.004 17433516

[B19] Szabo SJ Kim ST Costa GL Zhang X Fathman CG Glimcher LH A novel transcription factor, T-bet, directs Th1 lineage commitment Cell 2000 100 655 669 10.1016/S0092-8674(00)80702-3 10761931

[B20] Awasthi A Murugaiyan G Kuchroo VK Interplay between effector Th17 and regulatory T cells J Clin Immunol 2008 28 660 670 10.1007/s10875-008-9239-7 18810613

[B21] TomasiakŁozowska MM BodzentaŁukaszyk A Tomasiak M Skiepko R Zietkowski Z The role of interleukin 13 and interleukin 5 in asthma Postepy Hig Med 2010 64 146 155 20354262

[B22] Zhu W Liu N Zhao Y Jia H Cui B Ning G Association analysis of polymorphisms in IL-3, IL-4, IL-5, IL-9, and IL-13 with Graves' disease J Endocrinol Invest 2010 33 751 755 20332709 10.1007/BF03346682

[B23] Levine SJ Wenzel SE Narrative review: the role of Th2 immune pathway modulation in the treatment of severe asthma and its phenotypes Ann Intern Med 2010 152 232 237 20157138 10.1059/0003-4819-152-4-201002160-00008 PMC2846792

[B24] Pavón L Sandoval-López G Eugenia Hernández M Loría F Estrada I Pérez M Moreno J Avila U Leff P Antón B Heinze G Th2 cytokine response in Major Depressive Disorder patients before treatment J Neuroimmunol 2006 172 156 165 10.1016/j.jneuroim.2005.08.014 16457895

[B25] Cheng P Huang K Zhou G Zeng Z Wang T Li B Wang Y He L Feng G Shi Y Common SNPs in CSF2RB are associated with major depression and schizophrenia in the Chinese Han population World J Biol Psychiatry 2011 12 233 238 10.3109/15622975.2010.544328 21247258

[B26] Shelton RC Claiborne J Sidoryk-Wegrzynowicz M Reddy R Aschner M Lewis DA Mirnics K Altered expression of genes involved in inflammation and apoptosis in frontal cortex in major depression Mol Psychiatry 2011 16 751 762 10.1038/mp.2010.52 20479761 PMC2928407

[B27] Viinamäki H Heiskanen T Lehto SM Niskanen L Koivumaa-Honkanen H Tolmunen T Honkalampi K Saharinen T Haatainen K Hintikka J Association of depressive symptoms and metabolic syndrome in men Acta Psychiatr Scand 2009 120 23 29 10.1111/j.1600-0447.2008.01333.x 19133875

[B28] Honkalampi K Koivumaa-Honkanen H Lehto SM Hintikka J Haatainen K Rissanen T Viinamäki H Is alexithymia a risk factor for major depression, personality disorder, or alcohol use disorders? A prospective population-based study J Psychosom Res 2010 68 269 273 10.1016/j.jpsychores.2009.05.010 20159212

[B29] Beck AT Ward CH Mendelson M Mock J Erbaugh J An inventory for measuring depression Arch Gen Psychiatry 1961 4 561 571 10.1001/archpsyc.1961.01710120031004 13688369

[B30] American Psychiatric Association Diagnostic and Statistical Manual of Mental Disorders (4th edn) (DSM-IV) 1994 Washington, D.C.: American Psychiatric Publishing

[B31] First MB Spitzer RL Gibbon M Gibbon W Janet BW Structured Clinical Interview for DSM-IV-TR Axis I Disorders, Research Version, Non-patient Edition. (SCID-I/NP) 2002 New York: New York State Psychiatric Institute

[B32] First MB Gibbon M Spitzer RL Williams JBW Benjamin LS Structured Clinical Interview for DSM-IV Axis II Personality Disorders, (SCID-II) 1997 Washington, D.C.: American Psychiatric Press

[B33] Williams JBW Terman M Structured interview guide for the Hamilton Depression Rating Scale with Atypical Depression Supplement (SIGH-ADS) 2003 New York: New York State Psychiatric Institute

[B34] de Jager W te Velthuis H Prakken BJ Kuis W Rijkers GT Simultaneous Detection of 15 Human Cytokines in a Single Sample of Stimulated Peripheral Blood Mononuclear Cells Clin Diagn Lab Immunol 2003 10 133 139 10.1128/CDLI.10.1.133-139.2003 PMC145264 12522051

[B35] Elshal MF McCoy JP Multiplex bead array assays: performance evaluation and comparison of sensitivity to ELISA Methods 2006 38 317 323 10.1016/j.ymeth.2005.11.010 16481199 PMC1534009

[B36] Leng SX McElhaney JE Walston JD Xie D Fedarko NS Kuchel GA ELISA and multiplex technologies for cytokine measurement in inflammation and aging research J Gerontol A Biol Sci Med Sci 2008 63 879 884 10.1093/gerona/63.8.879 18772478 PMC2562869

[B37] Gottschalk PG Dunn JR The Five Parameter Logistic: A Characterization and Comparison with the Four-Parameter Logistic Analytical Biochemistry 2005 343 54 65 10.1016/j.ab.2005.04.035 15953581

[B38] Uh HW Hartgers FC Yazdanbakhsh M Houwing-Duistermaat JJ Evaluation of regression methods when immunological measurements are constrained by detection limits BMC Immunol 2008 9 59 10.1186/1471-2172-9-59 18928527 PMC2592244

[B39] González-Quintela A Vidal C Lojo S Pérez LF Otero-Antón E Gude F Barrio E Serum cytokines and increased total serum IgE in alcoholics Ann Allergy Asthma Immunol 1999 83 61 67 10.1016/S1081-1206(10)63514-4 10437818

[B40] Tsunoda M Tsunoda H Guevarra L Tollerud DJ The relation between serum cytokine levels and common laboratory tests in healthy Japanese males Environ Health Prev Med 2003 8 6 12 10.1007/BF02897937 21432109 PMC2723259

[B41] Hernández ME Mendieta D Martínez-Fong D Loría F Moreno J Estrada I Bojalil R Pavón L Variations in circulating cytokine levels during 52 week course of treatment with SSRI for major depressive disorder Eur Neuropsychopharmacol 2008 18 917 924 10.1016/j.euroneuro.2008.08.001 18805677

[B42] Simon NM McNamara K Chow CW Maser RS Papakostas GI Pollack MH Nierenberg AA Fava M Wong KK A detailed examination of cytokine abnormalities in Major Depressive Disorder Eur Neuropsychopharmacol 2008 18 230 233 10.1016/j.euroneuro.2007.06.004 17681762 PMC2267745

[B43] Bhakta NR Woodruff PG Human asthma phenotypes: from the clinic, to cytokines, and back again Immunol Rev 2011 242 220 232 10.1111/j.1600-065X.2011.01032.x 21682748 PMC3391010

[B44] Banks W Blood-Brain Barrier Transport of Cytokines: A Mechanism for Neuropathology Current Pharmaceutical Design 2005 11 973 984 10.2174/1381612053381684 15777248

[B45] Heim MH The Jak-STAT pathway: specific signal transduction from the cell membrane to the nucleus Eur J Clin Invest 1996 26 1 12 10.1046/j.1365-2362.1996.103248.x 8682149

[B46] Zambrano A Otth C Maccioni RB Concha II IL-3 control TAU modifications and protects cortical neurons from neurodegeneration Curr Alzheimer Res 2010 7 615 624 10.2174/156720510793499011 20964623

[B47] Hu F Pace TW Miller AH Interferon-alpha inhibits glucocorticoid receptor-mediated gene transcription via STAT5 activation in mouse HT22 cells Brain Behav Immun 2009 23 455 463 10.1016/j.bbi.2009.01.001 19167480 PMC2666112

[B48] Darnell JE Jr Kerr IM Stark GR Jak-STAT pathways and transcriptional activation in response to IFNs and other extracellular signaling proteins Science 1994 264 1415 1421 10.1126/science.8197455 8197455

[B49] Felger JC Alagbe O Hu F Mook D Freeman AA Sanchez MM Kalin NH Ratti E Nemeroff CB Miller AH Effects of interferon-alpha on rhesus monkeys: a nonhuman primate model of cytokine-induced depression Biol Psychiatry 2007 62 1324 1333 10.1016/j.biopsych.2007.05.026 17678633 PMC2149847

[B50] Yoshimura A Regulation of cytokine signaling by the SOCS and Spred family proteins Keio J Med 2009 58 78 83 10.2302/kjm.58.73 19597303

[B51] Phoenix TN Temple S Spred1, a negative regulator of Ras-MAPK-ERK, is enriched in CNS germinal zones, dampens NSC proliferation, and maintains ventricular zone structure Genes Dev 2010 24 45 56 10.1101/gad.1839510 20047999 PMC2802190

[B52] Denayer E Ahmed T Brems H Van Woerden G Borgesius NZ Callaerts-Vegh Z Yoshimura A Hartmann D Elgersma Y D'Hooge R Legius E Balschun D Spred1 is required for synaptic plasticity and hippocampus-dependent learning J Neurosci 2008 28 14443 14449 10.1523/JNEUROSCI.4698-08.2008 19118178 PMC6671253

[B53] Sutcigil L Oktenli C Musabak U Bozkurt A Cansever A Uzun O Sanisoglu SY Yesilova Z Ozmenler N Ozsahin A Sengul A Pro- and anti-inflammatory cytokine balance in major depression: effect of sertraline therapy Clin Dev Immunol 2007 7639 7646 10.1155/2007/76396 PMC2248234 18317531

[B54] KleinJan A Dijkstra MD Boks SS Severijnen LA Mulder PG Fokkens WJ Increase in IL-8, IL-10, IL-13, and RANTES mRNA levels (in situ hybridization) in the nasal mucosa after nasal allergen provocation J Allergy Clin Immunol 1999 103 441 450 10.1016/S0091-6749(99)70469-0 10069878

